# Monitoring the advancements in the technology of artificial cells by determining their complexity degree: Hints from complex systems descriptors

**DOI:** 10.3389/fbioe.2023.1132546

**Published:** 2023-02-01

**Authors:** Pier Luigi Gentili, Pasquale Stano

**Affiliations:** ^1^ Dipartimento di Chimica, Biologia e Biotecnologie, Università degli Studi di Perugia, Perugia, Italy; ^2^ Department of Biological and Environmental Sciences and Technologies (DiSTeBA), University of Salento, Ecotekne, Lecce, Italy

**Keywords:** artificial cells, complex systems, complexity, information theory, networks, protocells, synthetic biology, synthetic cells

## 1 Recent advancements in artificial cell technology pose new theoretical questions

In recent years an important momentum has characterized artificial cell (AC) research from the viewpoints of technical, conceptual, and functional achievements (Buddingh and van Hest, 2017; Salehi-Reyhani et al., 2017; Schwille et al., 2018; Stano, 2019; Abil and Danelon, 2020; Gaut and Adamala, 2021; Mukwaya et al., 2021; Staufer et al., 2021; Eto et al., 2022; Guindani et al., 2022). Although the ultimate goal of AC research–the construction of minimal *living* ACs from scratch–is still not so near^1^, the aforementioned advances raise an intriguing question. Is it possible to monitor these advancements by measuring AC “complexity”?

The inverted commas around the term complexity warn the readers that in this article, we use the term loosely, not claiming that ACs must exhibit, in their structure and/or behavior, the entire set of phenomena typically associated with Complex Systems *stricto sensu*. This sort of linguistic vagueness can be accepted, for the moment, just to start a discussion on AC complexity while keeping in mind the distinction between truly complex systems from just complicated ones^2^. The advantage of speaking of Complex Systems (CSs) is that it will permit – already from now – the application of tools and concepts taken from the information and computational theories employed for handling Natural Complexity so far.

With these warnings in mind, let us discuss possible approaches to define AC complexity and briefly illustrate a specific example. The goal of this Opinion paper is to attract attention to these intriguing topics and stimulate new discussions and proposals. We believe that broadening the field in this direction will increase the interest in AC technology, framing future developments in more engaging ways, and will contribute to finding a still missing universally accepted definition of Complex Systems.

## 2 Artificial cell complexity

The formal definition of CSs and a rigorous methodology for determining their degree of complexity are challenging tasks not yet accomplished (Mitchell 2009). A large number of definitions have been proposed in the literature: complexity measures actually abound (often referred to systems studied in physics) (Gell-mann & Lloyd 1996; Emmeche 1997; Lloyd 2001), but their relationship to biology is not always straightforward (Adami 2002).

Despite the lack of consensus on definitions and metrics, it can be agreed that any CS can be generally described as a network (Amaral and Ottino, 2004; Newman, 2011; Caldarelli, 2020; Gentili, 2021) whose constitutive components are nodes and links (Newman, 2010). The nodes are the elements of the network, whereas the links are the relationships among them. The network is intended as highly dynamic because CSs are maintained constantly out-of-equilibrium in the thermodynamic sense (Gentili 2018a). The strong interconnections among the nodes confer the power to show emergent properties to the network. Emergent properties belong to the network as a whole; they are collective and pop up through the non-linear integration of the nodes’ features (Anderson, 1972; Bar-Yam, 2004). Discriminating quantitatively the degree of complexity of distinct CSs is generally a daunting endeavor. As mentioned, this has been widely recognized by several authors, even reporting a large repertoire of complexity metrics used for disparate systems. A wise conclusion is probably that every specific problem is best described in a particular manner and that a proper complexity metric has to be correctly selected for each case.

### 2.1 Hierarchical analysis at three levels

How do the network perspectives described above apply to ACs? Any CS can be analyzed through three distinct hierarchical approaches: the reductionist, mesoscopic, and systemic ones (Gentili, 2021). The *reductionist* approach requires the determination of all the nodes and links that are the fundamental ingredients of any complex network. In a cell, the nodes are the molecules, and the links are the chemical reactions among them. The complexity of biological cells and ACs will depend on how many molecules and reactions are present, i.e., how many nodes and links constitute that particular network in a certain specific (topological) way. The reductionist approach might be reasonable in the case of ACs, while it is a daunting endeavor in the case of a living biological cell. An analysis of a cell at the *mesoscopic* level entails revealing the network “modules”. There are, e.g., signaling, genetic, and metabolic modules within a living cell. Then, the complexity of an AC can be determined by evidencing the number, the types, and the connection of modules, as well as their functions. Finally, ACs can be investigated at the *systemic* level. The systemic approach analyzes the functional features of the network as a whole. If we embrace the rationale of Natural Computing (Rozenberg et al., 2012; Gentili, 2018b), living cells and ACs can be conceived as “computing systems”. Any living cell has the power of encoding, collecting, storing, processing, and sending data and information to accomplish at the basic the purposes of surviving (self-maintenance) and reproducing (self-replication). If we assume any AC as a “computing” machine (for the moment devoted to some specific allopoietic task, but with the goal of being referred–in future–to autopoietic self-maintenance), then it is appropriate to pinpoint the number and kind of computations it makes, and its logic. In other words, it is valuable to determine the inputs it receives, the outputs it generates, and the corresponding computation. The larger the number of computations, the longer the corresponding algorithms, the higher its Kolmogorov complexity (Kolmogorov, 1968).

### 2.2 A case study inspired to the AC “bioreactor”

To briefly illustrate the above-mentioned approaches, we will apply them to a hypothetical AC inspired by the Noireaux and Libchaber (2004) “bioreactor”.

**FIGURE 1:**
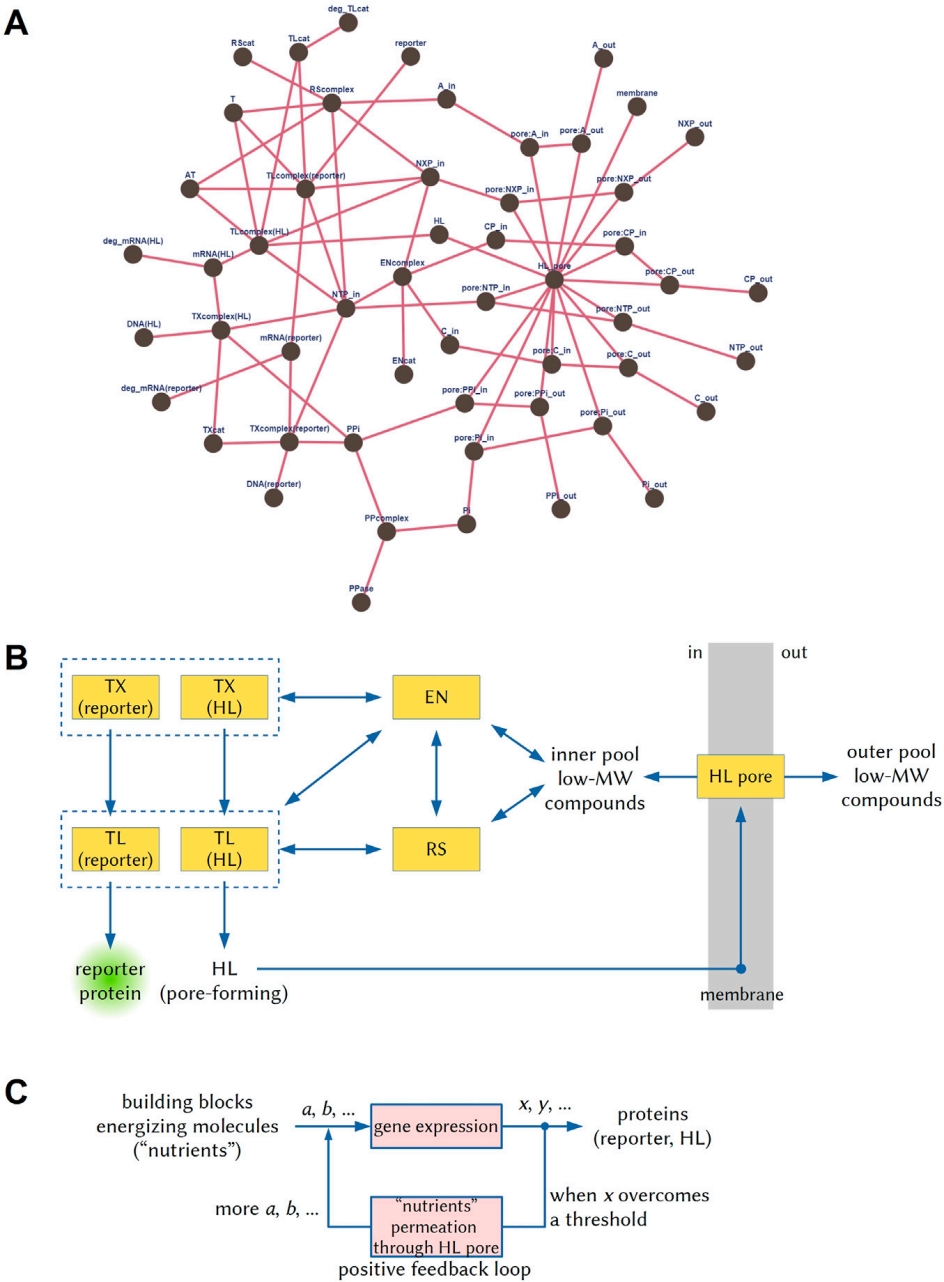
Graphical representations of the three hierarchical network-based approaches to describe ACs, i.e., the reductionist, mesoscopic, and systemic ones (Gentili, 2021). **(A)** The network of reactions occurring in the ACs drawn according to the reductionist approach, using the reaction set and the species described by (Mavelli et al., 2015) (details in the SI file). **(B)** The same network drawn according to the mesoscopic approach. TX: transcription; TL: translation; EN: energy recycling; RS: tRNA aminoacylation; HL: α-hemolysin; MW: molecular weight. **(C)** The systemic description refers instead to the network operations and their functional significance. In this case, *via* gene expression, a set of substrates (*a*, *b*, ...), which are amino acids, nucleotides triphosphate, *etc.*, are transformed into a set of products (*x*, *y*, ...), which include the reporter protein and the α-hemolysin. When the latter generate a membrane pore, a new process can take place, i.e., the diffusion of substrates from the external volume to the AC internal volume, leading to a sustained gene expression–in a sort of positive feedback loop.

In the *reductionistic* approach, the AC function needs to be described as a network of reactions. To this aim, it is first of all important to define at what degree of detail the network reactions must be described–several options are available. We opted for a coarse-grained description we previously developed to model intra-AC gene expression (Mavelli et al., 2015). It is detailed enough to represent key enzymes such as the RNA polymerase and the ribosome, but it groups together others (such as the several aminoacyl-tRNA synthetases, or the “energizing” enzymes). The passage of solutes through the α-hemolysin pore has been modeled as if the material exchange between the AC and its environment is mediated by a “universal” transporter (details and comments in the SI file). The resulting network is shown in Figure 1A, while the numerical values of some network metrics are given in the SI file. It results that the reductionistic approach allows a facile measurement of AC complexity, provided that the reactions involved in AC functioning can be described as a network according to a specific (agreed) level of description.

For the *mesoscopic* approach, the network of Figure 1A must be simplified, recognizing functional “modules”. Ideally, this should be done according to an objective procedure. A high number of links around some specific nodes suggests that those elements are at the modules’ core. It is easy to recognize transcription, translation, aminoacylation, energy regeneration, and transport modules, Figure 1B. This is not surprising because, actually, the coarse-grained model used for the reductionistic approach was originated by *thinking* in terms of modules. For a quantitative measure, the mesoscopic network can be analyzed as the reductionistic one, with the advantage that it is easier to define and can be therefore applied to more extended (and hence more complex) systems. It is interesting to note that while for some pivotal nodes (hubs) in Figure 1A (those with at least five links), a straight correspondence exists with Figure 1B modules, for others, it does not. This observation suggests that the reductionistic approach can reveal the presence of hubs impacting significantly on the whole AC functioning that are not intuitively recognized as modules. In this specific case, this unveiled role refers to intra-AC nucleotides triphosphates (NTP_in), and their partially dephosphorylated companions (NXP_in), both involved in energy-consuming processes and energy-recycling. This reveals the dual nature of these compounds, which participate in the network both as metabolic substrates and as energizing compounds.

Finally, Figure 1C shows a graphical description of the *systemic* approach, whereby the AC is described in terms of “what it does”, here translated into the language of computation and control. The graphical representation evidences the presence of a linear input-output process (whereby “output” products *x*, *y*, … are computed, i.e., produced, from “input” substrates *a*, *b*, … ) and of a *positive feedback loop* that functions at least for a certain time window (ideally: more α-hemolysin is produced, more pores are formed, more nutrients enter the AC and waste chemicals leave the AC, more proteins are synthesized). Considered as a whole, AC behaves under the control exerted by a natural computing device, which is the reaction network^3^. The net result of such computations can be described by an algorithm made of logical and operational instructions (see SI file). It can be written in different languages, as it happens in computer science, and its complexity can be quantified in terms of Kolmogorov complexity, related to its length (Kolmogorov, 1968). The AC complexity will correspond to the complexity of the algorithm that describes its functioning.

## 3 Defining and measuring AC complexity: An open question

It is evident that our discussion just scratches the surface of a challenging but stimulating problem: defining the complexity of the structured and functionally rich chemical systems we call ACs. It is worth noting that the issue of defining and measuring the complexity of ACs was briefly put forward in a previous publication (see SI file (Stano, 2019)), while in this Opinion piece, we have highlighted a hierarchical approach based on the reductionistic, mesoscopic and systemic network descriptors (Gentili, 2021). These approaches, we note, need a preliminary consensus on the definitions of the number and type of network elements (nodes and links) and the level-of-details of operational descriptions. For example, the nodes of reductionistic networks could refer to individual molecules or classes of molecules, to loosely or exactly defined complexes and reactions, to step-wise or all-in-one elongation processes of macromolecules, and so on. Similarly, the mesoscopic approach relies on the definition of what modules are and how to recognize them. The systemic approach instead needs the definition of a functional description of AC operations, opting for the most useful ones. Complexity metrics readily arise from these approaches, *given a set of agreed definitions*.

It should be noted that the network-based descriptions seem to put aside the notion of AC physical structure, at least in an explicit way. For example, the Noireaux and Libchaber (2004) bioreactor dynamics requires the presence of a membrane that separates two volumes, an α-hemolysin pore, and in/out molecular exchanges. These ingredients are fundamental, but they are only implicitly represented in Figure 1 (e.g., diffusible species are duplicated, labeled “in” and “out”). Adjacent or nested multicompartment ACs with coordinated dynamics, a hot topic in the field (Altamura et al., 2021), are clearly more complex than mono-compartment ones, but representations like the one of Figure 1A, although rigorous, just render the description of the system not easy to catch.

Several research articles have dealt with the problem of defining and measuring the complexity of organisms, and in general, of certain dynamical systems in biology (Hinegardner and Engelberg, 1983; McShea, 1996; 2000; Adami, 2002; Grizzi and Chiriva-Internati, 2005; Farnsworth et al., 2013; Bartlett and Beckett, 2019; Mayer, 2020; Rebout et al., 2021; Prinz, 2022). A detailed comment on the diverse approaches is not within the scope of this Opinion article. However, it is sufficient to mention that because current ACs are still far from being alive, thus being more machine-like than organism-like (Stano 2022), the problem generated by theoretical issues related to casting various definitions of complexity to current ACs is somehow simplified^4^.

Once a consensus definition of AC complexity and its measure will be achieved, how would they help AC research? Measuring AC complexity can help guide the experimental efforts in the direction of higher complexity, corresponding to more functional systems. Alternatively, complexity metrics can serve to minimize complexity, given a certain target behavior. AC technology, indeed, is a platform for investigating different questions, with different scopes and approaches. The resulting above-mentioned tension between moving to higher or lower complexity is only apparent. The complexity of the AC environment should be considered too. It will co-determine the AC complexity (in structure and organization) that allows an AC coping with it, in terms of the behavior that an AC must be able to perform to achieve specific goals (or, at least, not to stop functioning or to fall apart). This concept is reminiscent of Ashby law of “requisite variety” (Ashby, 1956). As we have remarked elsewhere (Damiano and Stano, 2018), looking at ACs as man-made synthetic biology systems from the perspective of early cyberneticians can open an interesting space of theoretical analyses, taking liberally on those pioneer ideas and conceptions.

## Footnotes

1 Building minimal *living* ACs from scratch challenges chemistry and biotechnology. Despite the recent progress, current ACs do not seem to be near the non-life to life threshold, and sometimes they are not even built for such a goal. Life-like features are often achieved in isolated manner (one or few at a time). Progress is however evident. On the other hand, practical scopes (that does not need living ACs) attract the interest and contribute to boost the field too (Leduc et al., 2007; Krinsky et al., 2018; Chang, 2019; Lussier et al., 2021; Sato et al., 2022).

2 Complex systems can be defined as those systems a) made up of complicated or interrelated parts; hard to separate, analyze, solve (Merriam-Webster’s Collegiate Dictionary, 2001); b) displaying distinct features such as chaotic behavior, emergent properties, computational intractability, and alike (Prokopenko et al., 2009). While definition a) corresponds to the common usage of the term “complexity”, definition b) refers to a precise technical meaning. The properties of a complicated system can be readily explained when the properties of the parts are known; *vice versa* the same is not true for truly complex systems exhibiting emergent properties, self-organization, adaptation, *etc.*

3 Such a device is ultimately embodied by the laws governing the chemical reactions in the network and the related chemicals (reactants, templates, catalysts, inhibitors, *etc.*), i.e., molecular recognition, reaction connections, binding and kinetic constants.

4 The machine/organism duality (as well as the mind/computer one) has generated a long-standing debate that is far from being settled (Carello et al., 1984; Danchin, 2009; Shapiro, 2012; Nicholson, 2013; Boldt, 2018), which refers to our fundamental understanding of what life (or intelligence) is. Just to give an example, it has been suggested that the self-referentiality typical of biological organisms is equivalent to stating their non-computability by a Turing machine (Letelier et al., 2003; McMullin, 2004).
