# Supplementary material for: Monitoring the advancements in the technology of artificial cells by determining their complexity degree: Hints from complex systems descriptors
Source: Front Bioeng Biotechnol. 2023 Feb 1;11:1132546. doi: 10.3389/fbioe.2023.1132546 (PMC9928734; doi:10.3389/fbioe.2023.1132546)
Supplement: Supplementary file 1 [file DataSheet1.PDF]

Supplementary Material

1 AN EARLY DISCUSSION ON ARTIFICIAL CELL COMPLEXITY [11]

Structural complexity accounts for: (a1) the number of different molecules and their numerousness: the chemical composition of an AC could be displayed in a ranking plot, and the shape of the resulting distribution could be evaluated by quantitative analysis; (a2) a quantitative measurement of the information required to specify a certain structure, in the sense illustrated by [2], and as proposed by [5]. It should be noted that structural complexity is best defined as scale-independent metrics, i.e., not depending on the AC size (by applying a sort of size-normalization factor). *Organizational (or functional) complexity* accounts for: (b1) the reactions occurring inside SCs, schematically shown as a network, whose complexity could be measured by known metrics; (b2) the AC behavior, represented as the shortest algorithm that would be able to generate a computer model of the AC. The corresponding algorithm complexity becomes a proxy for the AC complexity. Possible metrics are the algorithm length (Kolmogorov-Solomonoff-Chaitin complexity, [6]), or the cyclomatic complexity – i.e., the number of different paths that an algorithm can take [9].

2 DETAILS ABOUT THE NETWORK DRAWN IN FIGURE 1A

The network drawn in **Figure 1A** (according to the reductionist approach) represents the topology of a set of reactions occurring in an Artificial Cell (AC) inspired to the Noireaux and Libchaber bioreactor [10]. In particular, the AC hosts a transcription (TX) – translation (TL) reaction system that reads two DNA sequences and produces, respectively, a reporter fluorescent protein and α-hemolysin. The latter self-assembles on the membrane as an heptameric pore that allows the passage of low-MW molecules from the external environment to the AC lumen and vice versa. Molecules that are necessary to the reactions are indeed present in the AC environment in order to feed the intra-AC reactions [10].

Although the approach we are referring to (**Figure 1A**) has been called "reductionist", a detailed description of *all* reactions occurring in a TX-TL system (including the elongation steps in RNA and protein synthesis) is very difficult. We opted, therefore, for a coarse-grained model we recently developed to study protein synthesis dynamics and stochastic solute encapsulation [8, 7, 3]. The model was developed on the basis of a previous work [12]. In the context of determining the complexity metrics in AC research, deciding at what degree of details a reductionist network should be drawn is a matter of agreed convention.

As explained in [7], TX-TL reactants and catalysts (the enzymes) are grouped in pseudo-species. For example, "NTP" represents all types of nucleotide triphosphates, "AT" represents all aminoacylated tRNAs, "TLcat" represents the ribosomes and translation factors required for translation, "a" represents polymerized amino acids (i.e., the produced protein), "T" represents all tRNAs, "NXP" represents all types of dephosphorylated nucleotide triphosphates, etc. The reactions are described by including substrate-catalyst complexes explicitly. For example, the translation (TL) reaction is represented as:

$$\mathrm{AT} + \mathrm{NTPin} \xrightarrow[\text{RNA}]{\text{TLcat}} \mathrm{a} + \mathrm{T} + \mathrm{NXPin}$$

Once all reactions occurring in the TX-TL systems have been defined, the system dynamics can be simulated by numerically solving the set of differential equations that describe the rates of the chemical reactions and of the diffusion flows occurring in the AC/environment super-system. In [7], reaction rates

have been expressed as Michaelis-Menten functions. To draw the network (**Figure 1A**), however, it is sufficient to establish the connectivity between the chemical species involved in each process (reactants, catalysts, intermediates, products) and consequently generate the so-called *adjacency matrix* (a square matrix used to represent a finite graph; the elements of the matrix, 1 or 0, indicate whether pairs of vertices are adjacent or not in the graph [1]). The network is the graphical representation of the adjacency matrix.

For example, the above-mentioned translation reaction contributes with two links to the network: the first refers to the formation of the substrate(s)-enzyme-template complex $\mathrm{C_{TL}}$, the second refers to its breakdown, leading to products:

$$\mathrm{AT + NTPin + TLcat + RNA \rightleftharpoons C_{TL} \longrightarrow a + T + NXPin + Pi + TLcat + RNA}$$

To model the diffusion of small-MW compounds through the α-hemolysin pore we treat the latter as a passive universal transporter. The diffusive process of a given species S through the pore has been modeled as it follows:

$$\mathrm{pore + Sout \rightleftharpoons pore{:}Sout \rightleftharpoons pore{:}Sin \rightleftharpoons pore + Sin}$$

where the two central species (pore-Sout and pore-Sin) represent, respectively, the species S “bound” to the outer and inner face of the pore. In other words, the process has been made discrete, and can be represented, in **Figure 1A**, with three consecutive links. Although it does not provide a physically accurate description of the concentration-driven diffusion, this escamotage allows a representation of small-MW compound diffusion in form of reactions – which can be integrated with the others in the network. Again, for the sake of drawing chemical networks for the scope described in this article, the choice should be functional to the scope, and the degree of details comes from an agreed convention, or at least fixed once for all when the aim is a comparative one. Note that the compartmentalized nature of the network in **Figure 1A**, and therefore the division of the AC/environment super-system volume in two parts (inner and outer one) is only implicitly represented; in particular, it is deduced by the “in” and “out” subscripts attached to some species (to avoid extensive labeling, species with no subscripts are intended as localized inside the AC).

3 NETWORK METRICS

Once a network that describes the AC processes has been determined, it is possible to “measure” its structure (complexity) by using various metrics that have been developed in network and graph theories. Some of the most common ones are listed in Table S1.

Additional metrical values can be obtained by considering the distribution of the connectivity degree of the network nodes. The degree distribution of the Figure 1A network is shown in Figure S1, from which it is possible to calculate the average degree $\overline{d}$, i.e., the average number of neighbors:

$$\overline{d} = \frac{\sum_i n_i d_i}{N} = 2.94$$

where n_i is the number of nodes having degree d_i; N is the total number of nodes.

The entropy of the network can be calculated through the Shannon’s formula:

Table S1. Values of some network metrics referred to the network in Figure 1A of the main text.

Network Metrics	Value
Number of nodes	53
Number of links	78
Network diameter[1]	7
Network radius	4
Characteristic path length	3.486
Clustering coefficient	0.089
Network density	0.057
Network heterogeneity	0.831
Network centralization	0.261

[1] The shortest distance between the two most distant nodes in the network.

$$H = -\sum_i f_i \log_2 f_i = -\frac{\sum_i f_i \log_{10} f_i}{\log_{10} 2} \approx -3.32 \sum_i f_i \log_{10} f_i = 2.24 \text{ bits}$$

Since the network consists of 53 nodes, the largest Shannon entropy possible is when $f_i = 1/53 \; \forall i$, and it is $H = 5.72$ bits.

Alternatively, the normalized Network Entropy [4] can be calculated through the following equation:

$$H_n = \frac{1}{N \ln (N-1)} \sum_{i=1}^{N} \ln d_i = 0.048$$

The normalized Network Entropy is maximal and equal to 1 for fully connected networks. The sparser the network, the lower becomes its Network Entropy.

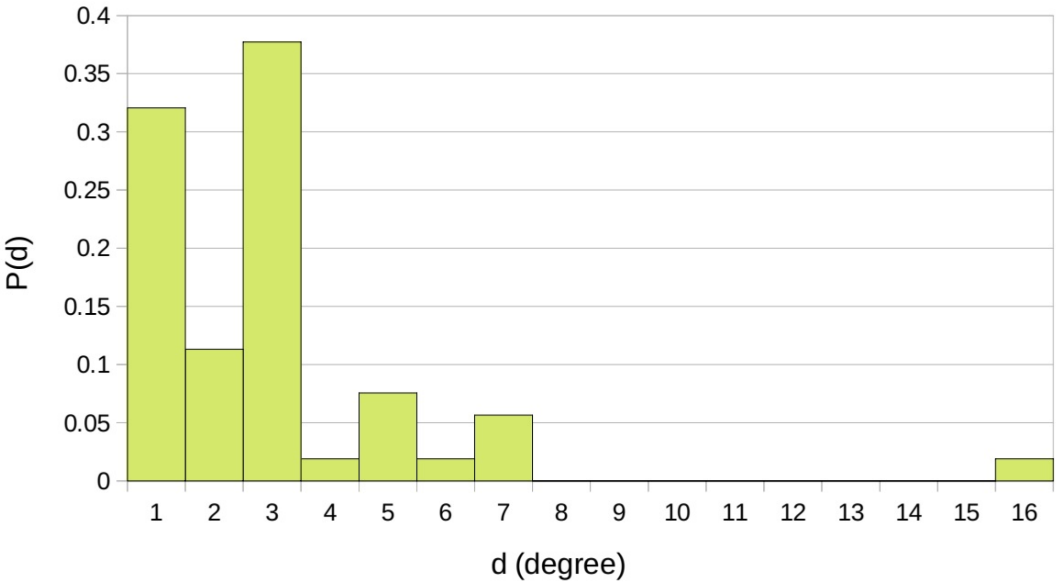

Figure S1. Plot of the degree distribution for the network represented in Figure 1A

4 ALGORITHMIC DESCRIPTION

If the AC behavior is described by an algorithm, the algorithm complexity can be considered as a proxy for AC complexity. The algorithm will describe the operations carried out by the AC. In other words, ACs are considered machines whose behavior can be described by a list of instructions. For example, the algorithm can be used to generate a computer model of the physical AC. As mentioned above, a classical definition of complexity refers to the length of the shortest program that produces the target behavior [6]. Another metrics could be the “cyclomatic complexity”, which refers to the number of linearly independent paths in an algorithm [9].

The algorithmic description can be based on standard logical operators like “IF... THEN” decision structures, “FOR” iterations, etc. Operations, however, can be formulated at different degrees of details (as it happens in formal languages used to code). Therefore, this approach also needs an agreed definition of which formal “language” should be firstly developed (obeying to proper logical requisites) and later used. Hints could come from languages currently adopted in systems biology.

For the showcased AC, a possible algorithmic description of its behavior is tentatively given by the script below, written in form of a pseudo-code.

SET time[initial time, end time]
SET outer pool of low-MW compounds[A_{out}, NTP_{out}, ...]
SET inner pool of low-MW compounds[A_{in}, NTP_{in}, ...]
SET genes[hemolysin, reporter]
SET mRNAs[RNA_{hem}, RNA_{rep}]
SET macromolecular machinery[TXcat, TLcat, ...]
SET produced proteins[α-hemolysin, reporter, pore]

FUNCTION gene expression
(*calculation of protein concentration over time, based on the concentrations of inner compounds such as the DNA template, the catalysts, the amino acids, nucleotides, tRNAs, and according to a complex kinetic scheme that involved transcription, translation, aminoacyl-tRNA synthesis, energy recycling*)
FUNCTION degradation
(*calculation of the amount of degraded (inactive) mRNAs and macromolecular machineries, following a slow unimolecular processes*)
FUNCTION pore assembly
(*calculation of the number of α-hemolysin pores – as heptamers – based on the value of α-hemolysin concentration and a critical value $C_{critical}$*)
FUNCTION diffusion
(*calculation of the concentration of low-MW molecules given a diffusion across the membrane – driven by the concentration gradient, according the number of α-hemolysin pores, if available*)

START

FOR time = initial time **TO** time = end time
DO gene expression
DO degradation

IF produced protein[α-hemolysin] $> C_{critical}$ **THEN**
DO pore assembly

IF produced protein[pore] > 0 **THEN**
DO diffusion
ENDFOR

END

REFERENCES

[1]N. Biggs. *Algebraic Graph Theory*. Cambridge Mathematical Library. Cambridge University Press, 2nd edition, 1993.

[2]L. Brillouin. *Science and Information Theory*. Academic Press, Inc., New York, 2nd edition, 1962.

[3]P. Carrara, E. Altamura, F. D'Angelo, F. Mavelli, and P. Stano. Measurement and Numerical Modeling of Cell-Free Protein Synthesis: Combinatorial Block-Variants of the PURE System. *Data*, 3(4):41, 2018.

[4]C. G. S. Freitas, A. L. L. Aquino, H. S. Ramos, A. C. Frery, and O. A. Rosso. A detailed characterization of complex networks using Information Theory. *Sci Rep*, 9(1):16689, 2019.

[5]Y. Jiang and C. Xu. The calculation of information and organismal complexity. *Biol Direct*, 5:59, Oct. 2010.

[6]A. N. Kolmogorov. Three approaches to the quantitative definition of information. *International Journal of Computer Mathematics*, 2(1-4):157–168, 1968.

[7]F. Mavelli, R. Marangoni, and P. Stano. A Simple Protein Synthesis Model for the PURE System Operation. *Bull. Math. Biol.*, 77(6):1185–1212, 2015.

[8]F. Mavelli and P. Stano. Experiments on and Numerical Modeling of the Capture and Concentration of Transcription-Translation Machinery inside Vesicles. *Artif. Life*, 21(4):445–463, 2015.

[9]T. J. McCabe. A complexity measure. *IEEE Trans. Soft. Eng.*, SE-2(4):308–320, 1976.

[10]V. Noireaux and A. Libchaber. A vesicle bioreactor as a step toward an artificial cell assembly. *Proc. Natl. Acad. Sci. U.S.A.*, 101(51):17669–17674, 2004.

[11]P. Stano. Is Research on "Synthetic Cells" Moving to the Next Level? *Life*, 9(1):3, 2019.

[12]T. Stögbauer, L. Windhager, R. Zimmer, and J. O. Rädler. Experiment and mathematical modeling of gene expression dynamics in a cell-free system. *Integr. Biol.*, 4(5):494–501, 2012.
